# Molecular characterization of root-knot nematodes (*Meloidogyne* spp.) from Arkansas, USA

**DOI:** 10.1038/s41598-019-52118-4

**Published:** 2019-10-30

**Authors:** Weimin Ye, Robert Thomas Robbins, Terry Kirkpatrick

**Affiliations:** 1 Nematode Assay Section, Agronomic Division, North Carolina Department of Agriculture & Consumer Services, Raleigh, North Carolina 27607 United States of America; 2 Department of Plant Pathology, Plant Sciences Building 217A, University of Arkansas, Fayetteville, Arkansas 72701 United States of America

**Keywords:** Molecular biology, Plant sciences

## Abstract

Root-knot nematodes (*Meloidogyne* spp.) are the most common major pathogens of many crops throughout the world, impacting both the quantity and quality of marketable yields. In this study, a total of 244 root-knot nematode populations from various hosts from 39 counties in Arkansas were tested to determine the species diversity. Molecular characterization was performed on these populations by DNA sequencing of the ribosomal DNA 18S-ITS-5.8S, 28S D2/D3 and a mitochondrial DNA fragment flanking cytochrome oxidase gene subunit II - the intergenic spacer. Five species were identified, including *M. incognita* (Kofoid & White, 1919) Chitwood, 1949 from soybean, cotton, corn and various vegetables (232 samples); *M. hapla* Chitwood, 1949 from rose (1 sample); *M. haplanaria* Eisenback, Bernard, Starr, Lee & Tomaszewski, 2003 from okra, tomato, peanut, Indian hawthorn, ash, willow and elm trees (7 samples); *M. marylandi* Jepson & Golden in Jepson, 1987 from grasses (3 samples); and *M. partityla* Kleynhans, 1986 from pecan (1 sample) through a combined analysis of DNA sequencing and PCR by species-specific primers. *Meloidogyne incognita* is the most abundant species that was identified in 95% samples and was the only species in field crops including soybean and cotton, except for one population of *M. haplanaria* from soybean in Logan County (TK201). Species-specific primers were used to verify *M. incognita* through PCR by species-specific primers. Unlike historical data, *M. arenaria*, *M. javanica* and *M. graminis* were not detected from any of the samples collected during this study. This result is essential for effective and sustainable management strategies against root-knot nematodes in Arkansas.

## Introduction

Root-knot nematodes (RKN) are microscopic worms that live in soil and feed on the roots of many crops and weeds. The nematode gets its name because its feeding causes galls to form on the roots of infected plants. They are sedentary endoparasitic nematodes that depend on the induction of a permanent feeding site in living roots to complete their life cycle. RKN are the most widespread and serious plant-parasitic nematode pests, damaging a very wide range of crops throughout the world^1^. They are scientifically classified in the genus *Meloidogyne* (Tylenchida: Meloidogynidae) with over 100 species described^2^.

The southern RKN, *M. incognita* (Kofoid & White) Chitwood, 1949, is the most important nematode parasite of cotton in Arkansas^3^ and it has replaced the soybean cyst nematode as the premier nematode pest of soybean^4^. RKN are also commonly found in corn and grain sorghum fields and are associated with various horticultural and ornamental crops and turf grasses in the state. Because of the significance of the agricultural production to Arkansas’ economy^5^, understanding the *Meloidogyne* species associated with crops in the state is vital to formulation of effective and sustainable management strategies.

Previous surveys of RKN in Arkansas were conducted by using classical morphological methods. In a few surveys from soybean^6^, cotton^7^, wheat^8^ and blueberry^9^, RKN were found but species identification was not attempted. *Meloidogyne graminis* (Sledge & Golden, 1964) Whitehead, 1968 was first found in 1967 by R. D. Riggs on *Zoysia* spp. in Arkansas^10^. *Meloidogyne hapla* Chitwood, 1949 was reported on black locust (*Robinia pseudoacacia*) near the Mississippi River in Arkansas^11^. Norton *et al*.^12^ documented the occurrence of *M. arenaria* (Neal, 1889) Chitwood, 1949, *M. hapla*, and *M. incognita* in Arkansas. Wehunt *et al*.^13^ reported *M. incognita*, *M. hapla*, *M. arenaria*, *M. graminis*, and *M. javanica* from soybean fields near the Mississippi river. Elmi *et al*.^14,15^ recorded *M. marylandi* Jepson & Golden in Jepson, 1987 from tall fescue. Walters and Barker^16^ reported *M. hapla*, *M. incognita*, *M. arenaria*, and *M. javanica* (Treub, 1885) Chitwood, 1949 in Arkansas. In a recent survey from 106 soil and root samples, *M. incognita*, *M. marylandi, M. haplanaria, M. hapla, M. arenaria* and *M. partityla* Kleynhans, 1986 were identified through molecular diagnosis and *M. incognita* was the most abundant species^17^.

Development of resistant varieties that suppress nematode growth and reproductions is the most desirable, cost-effective and environmentally sustainable strategy for managing plant-parasitic nematodes^18^. Host plant resistance is effective against certain species or races; thus, accurate identification of RKN species is critical to the success of the use of host resistance or rotation. Species of RKN has been traditionally identified based on female perineal pattern, second-stage juvenile and male morphology and morphometrics, isozyme analysis, and host differential test. The traditional methods are always challenging due to highly conserved and similar morphology across species, lack of certain life stages, high intraspecies variability, potential hybrid origin and polyploidy^19^. In the past 20 years, molecular tools have been progressively developed to identify RKN species using polymerase chain reaction (PCR), Restriction Fragment Length Polymorphism (RFLP), and DNA sequencing, because they are usually fast, sensitive, less subjective and applicable to any life stages of a population^19–25^. The objective of this study was to collect RKN samples from field crops and natural sites in the state of Arkansas and to characterize the DNA sequences of RKN on the ribosomal DNA 18S-ITS-5.8S, 28S D2/D3 and mitochondrial DNA cytochrome oxidase gene subunit II-the intergenic spacer (CoxII-IGS) to determine the species and their distribution.

## Results

### RKN problem in Arkansas

**Figure 1:**
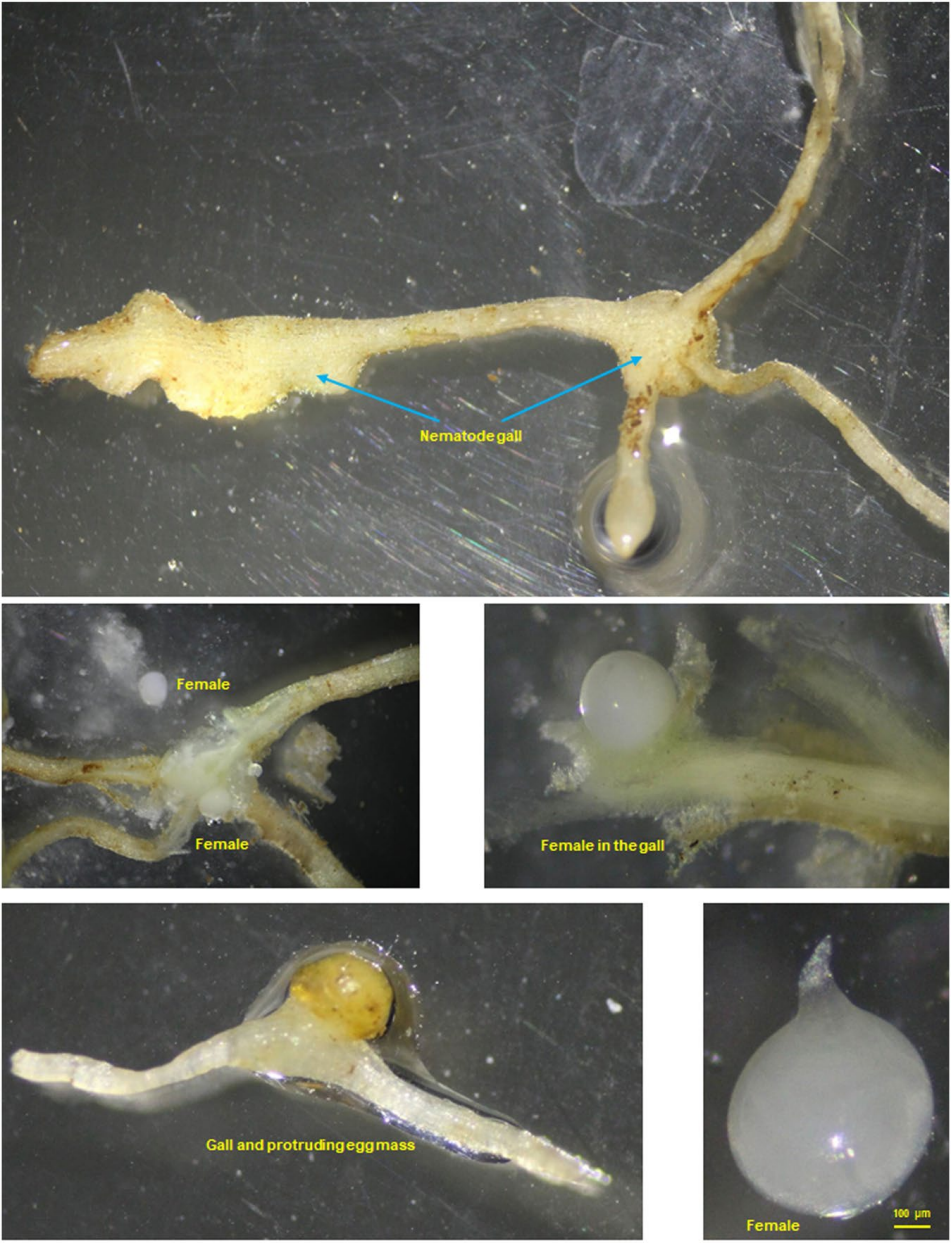
Photographs of root galls and females of southern root-knot nematode (*Meloidogyne incognita*) from tomato in Pulaski, Arkansas (RT122).

**Figure 2:**
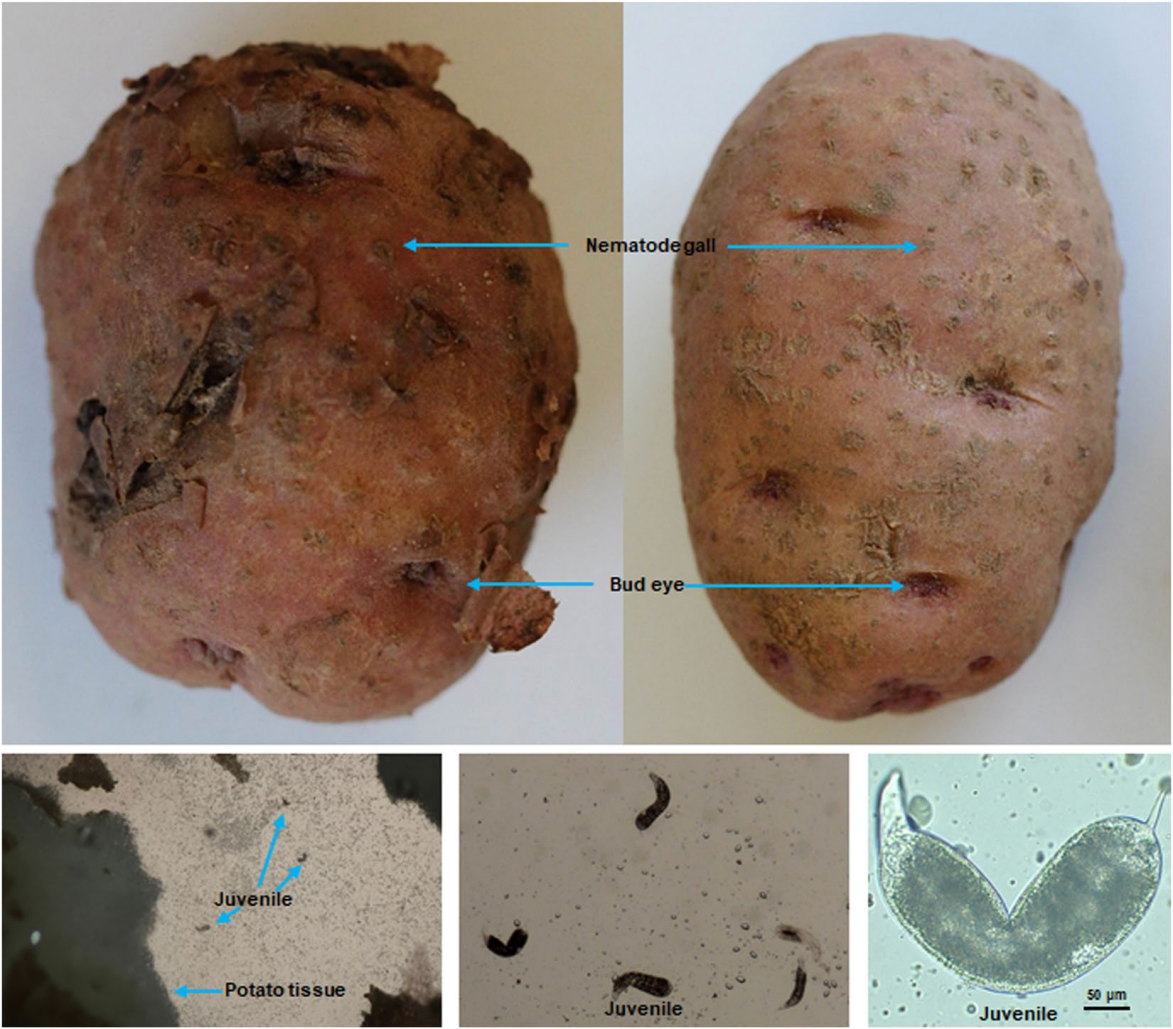
Photographs of the infested potato and the juveniles of southern root-knot nematode (*Meloidogyne incognita*) from potato in Van Buren County, Arkansas (RT139).

**Figure 3:**
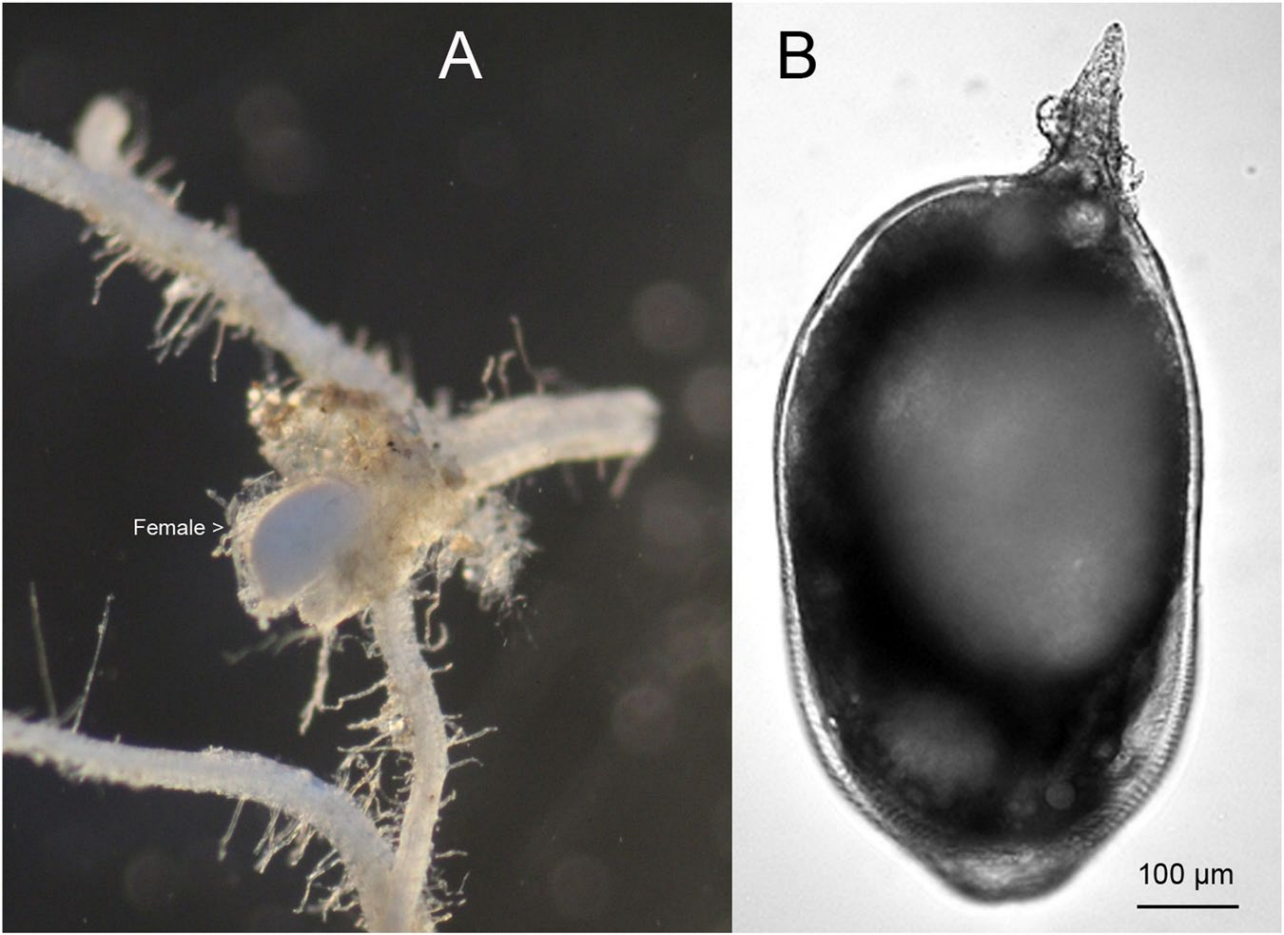
Photographs of females of Maryland root-knot nematode (*Meloidogyne marylandi*) from Sedge like grass in Washington County, Arkansas (RT106). (**A**) Female on the root. (**B**) Female.

RKN are common in field samples submitted to the Arkansas Nematode Diagnostic Laboratory. Infected roots have typical gall formation and RKN females, juveniles and egg masses could be recovered from the galled tissues (Figs 1 and 2). *Meloidogyne marylandi* does not produce galls on turfgrasses, and only semi-penetrates the roots (Fig. 3A). The female is lemon-shaped, with a much harder cuticle and a slightly protruding vulva-anus region (Fig. 3B), that is different from the pear-shaped female and rounded vulva-anus region in other common RKN living inside the galls (Fig. 1).

### RKN identification

**Figure 4:**
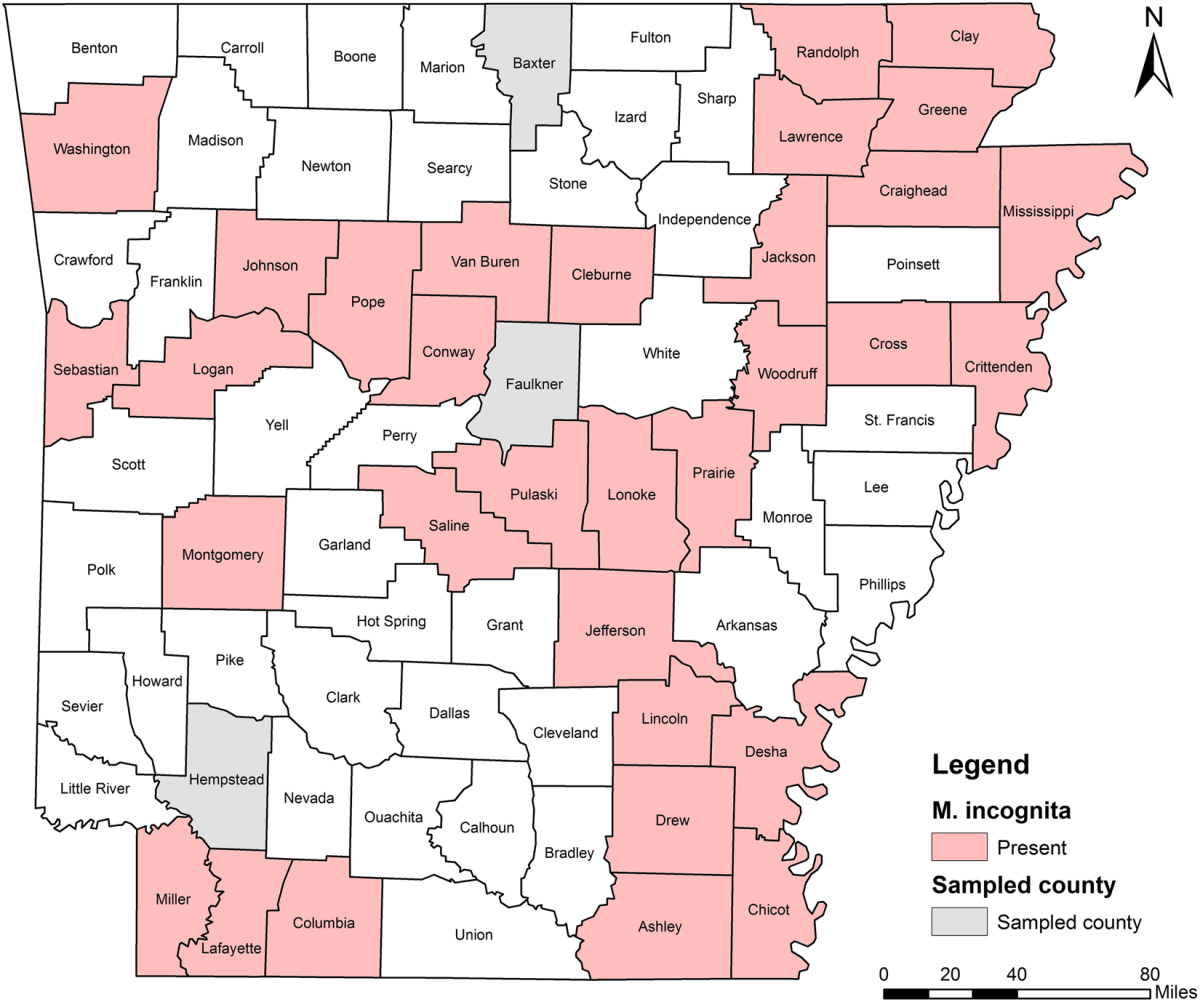
Distribution of southern root-knot nematode (*Meloidogyne incognita*) in Arkansas.

**Figure 5:**
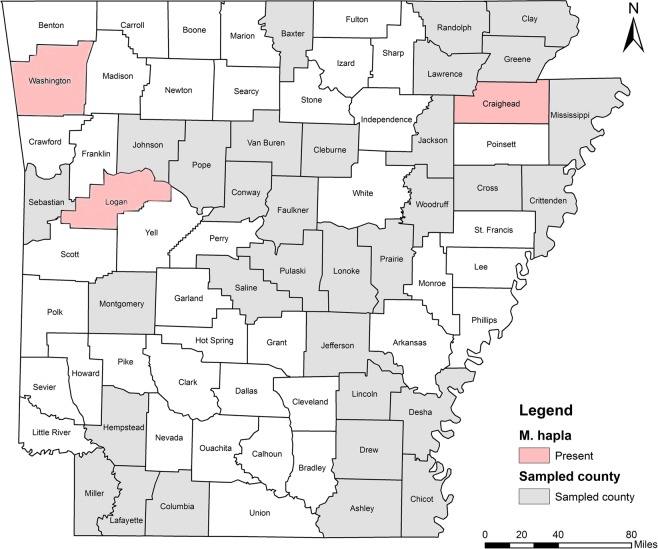
Distribution of Texas peanut root-knot nematode (*Meloidogyne haplanaria*) in Arkansas.

**Figure 6:**
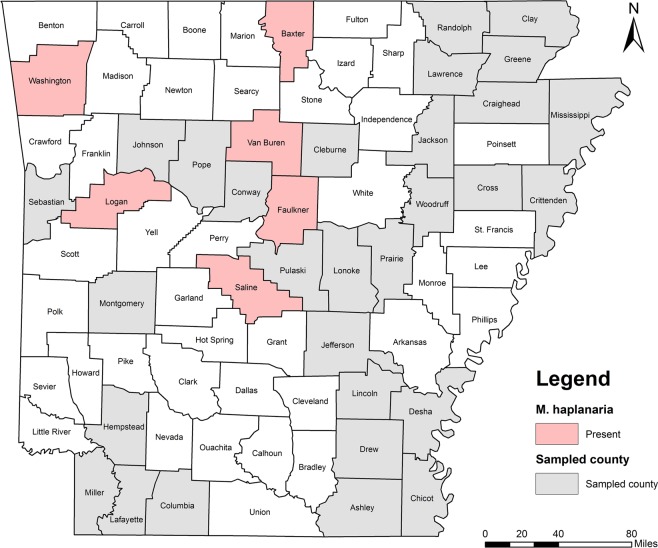
Distribution of northern root-knot nematode (*Meloidogyne hapla*) in Arkansas. Logan and Washington counties were from results by Khanal *et al*.^17^.

**Figure 7:**
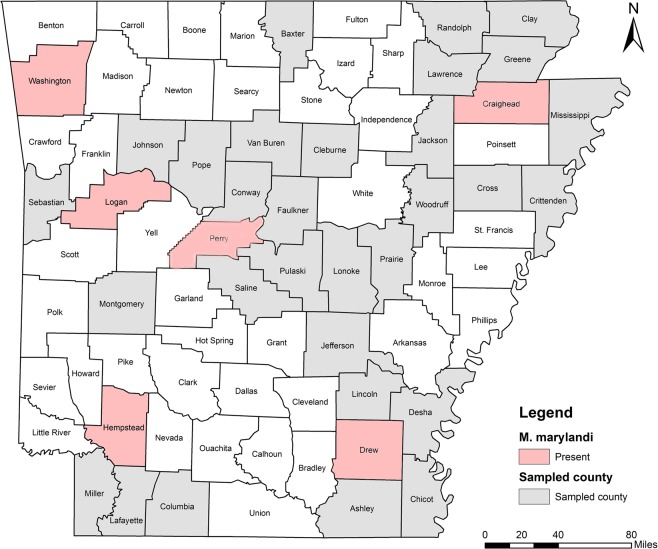
Distribution of Maryland root-knot nematode (*Meloidogyne marylandi*) from Arkansas. Drew, Craighead and Perry counties were from results by Khanal *et al*.^17^.

**Figure 8:**
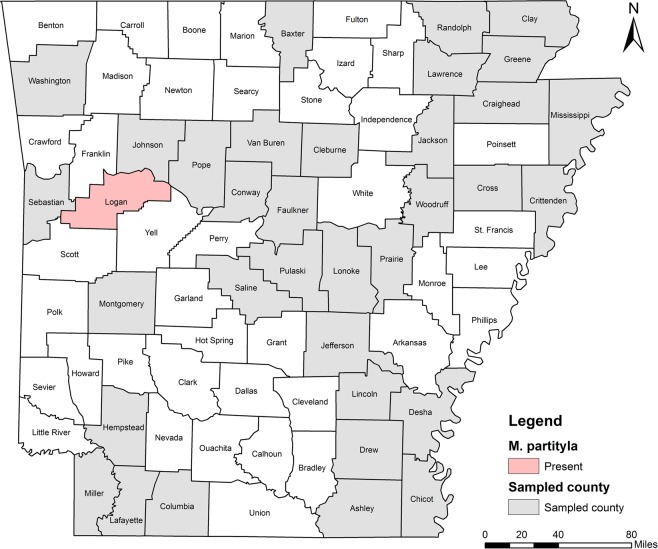
Distribution of pecan root-knot nematode (*Meloidogyne partityla*) from Arkansas.

Five RKN species were identified including *M. incognita*, *M. hapla*, *M. haplanaria*, *M. marylandi* and *M. partityla*; the results are presented in Table 1. Species identification in this study was based on the combined analysis of DNA sequencing on the rDNA 18S-ITS-5.8S, 28S D2/D3 and CoxII-IGS (Table 1) and PCR by species-specific primers (Table 2). *Meloidogyne incognita*, the most prevalent species, was found in 232 samples (95%) from soybean, cotton, corn and various vegetables in 36 of the 39 counties from which samples were collected (Ashley, Bradley, Chicot, Clay, Cleburne, Columbia, Conway, Craighead, Crittenden, Cross, Desha, Drew, Garland, Greene, Jackson, Jefferson, Johnson, Lafayette, Lawrence, Lincoln, Logan, Lonoke, Miller, Mississippi, Montgomery, Phillips, Pope, Prairie, Pulaski, Randolph, Saline, Sebastian, Van Buren, Washington, Woodruff, and Yell) (Fig. 4). *Meloidogyne hapla* was found in only one sample from rose in Craighead County (Fig. 5). *Meloidogyne haplanaria* was found in seven samples from okra, tomato, peanut, Indian hawthorn, ash, willow and elm trees in Baxter, Faulkner, Logan, Saline, Van Buren, and Washington counties (Fig. 6). *Meloidogyne marylandi* was found in three samples from grasses in Hempstead, Logan, and Washington counties (Fig. 7). *Meloidogyne partityla* was found in only one sample from pecan in Logan County (Fig. 8). There were no samples with mixtures of species found.

**Table 1:** Species and isolates of root-knot nematodes (*Meloidogyne* spp.) sequenced in the present study.

No.	DNA ID	Species	Host	County	18S + ITS GenBank Accession No.	28S D2/D3 GenBank Accession No.	CoxII-IGS GenBank Accession No.
1	RT70	*M. incognita*	Tomato	Pulaski		MK102787	MK102799
2	RT73	*M. incognita*	Cucumber on Tomato	Pulaski		MK102787	MK102799
3	RT75	*M. incognita*	Soybean	Drew		MK102787	MK102798
4	RT76	*M. haplanaria*	Ash	Washington	MK102773	MK102784	MK102794
5	RT77	*M. incognita*	Cucumber	Pulaski	MK102776		MK102799
6	RT78	*M. incognita*	Tomato	Sebastian	MK102776	MK102787	MK102799
7	RT79	*M. incognita*	Okra	Pulaski	MK102776	MK102787	MK102800
8	RT80	*M. incognita*	Tomato	Pulaski	MK102778	MK102787	MK102799
9	RT81	*M. incognita*	Pocket melon	Pulaski	MK102775	MK102787	MK102799
10	RT82	*M. haplanaria*	Okra	Van Buren	MK102773	MK102784	MK102794
11	RT83	*M. hapla*	Knockout rose	Craighead	MK102780	MN475814	MK102792
12	RT84	*M. incognita*	Carrot	Washington	MK102778	MK102787	MK102799
13	RT85	*M. haplanaria*	Tomato	Baxter	MK102778		MK102794
14	RT97	*M. marylandi*	Italian rye grass	Logan	MK102774	MK102782	MK102797
15	RT98	*M. incognita*	Tomato	Logan	MK102776	MK102787	
16	RT99	*M. incognita*	Soybean on Tomato	Woodruff	MK102776	MK102787	MK102799
17	RT100	*M. incognita*	Soybean	Saline	MK102778	MK102787	
18	RT101	*M. haplanaria*	Peanut	Saline	MK102772	MK102785	MK102794
19	RT102	*M. incognita*	Fig	Pulaski	MK102776	MK102787	
20	RT106	*M. marylandi*	Sedge like grass	Washington		MK102782	
21	RT118	*M. incognita*	Holy basil	Montgomery	MK102778	MK102790	MK102799
22	RT120	*M. incognita*	Pinto bean	Conway		MK102787	MK102799
23	RT121	*M. incognita*	Tomato	Pope	MK102776	MK102787	MK102799
24	RT122	*M. incognita*	Tomato, okra	Pulaski	MK102778	MK102787	
25	RT126	*M. incognita*	Soybean	Woodruff	MK102776		
26	RT127	*M. incognita*	Zucchini	Washington	MK102776		
27	RT128	*M. partityla*	Pecan	Logan		MK102783	MK102796
28	RT129	*M. marylandi*	Bermuda grass	Hempstead	MK102781		
29	RT130	*M. haplanaria*	Willow, elm	Washington	MK102772		MK102795
30	RT131	*M. incognita*	Tomato	Bradley			KU948024
31	RT132	*M. incognita*	Squash	Cleburne			KU948016
32	RT133	*M. incognita*	Tomato	Columbia			KU948016
33	RT134	*M. haplanaria*	Indian hawthorn	Faulkner			KU948026
34	RT135	*M. incognita*	Okra	Garland			KU948025
35	RT136	*M. incognita*	Soybean	Logan			KU948016
36	RT137	*M. incognita*	Squash, cucumber	Phillips			KU948021
37	RT138	*M. incognita*	Soybean	Yell			KU948016
38	RT139	*M. incognita*	Potato	Van Buren	MK102778	MK102787	
39	TK1	*M. incognita*	Soybean	Lonoke	MK102777	MK102787	
40	TK2	*M. incognita*	Corn	Desha	MK102776	MK102787	
41	TK3	*M. incognita*	Corn	Desha		MK102787	
42	TK4	*M. incognita*	Corn	Desha		MK102787	
43	TK5	*M. incognita*	Cotton	Desha	MK102778	MK102787	
44	TK6	*M. incognita*	Soybean	Lincoln		MK102787	MK102799
45	TK7	*M. incognita*	Soybean	Lincoln	MK102778	MK102787	
46	TK8	*M. incognita*	Soybean	Desha	MK102778	MK102787	
47	TK9	*M. incognita*	Soybean	Desha	MK102778	MK102787	MK102799
48	TK10	*M. incognita*	Soybean	Desha		MK102787	
49	TK11	*M. incognita*	Soybean	Mississippi	MK102778	MK102787	MK102799
50	TK12	*M. incognita*	Soybean	Mississippi		MK102787	MK102799
51	TK13	*M. incognita*	Corn	Mississippi	MK102777	MK102787	
52	TK14	*M. incognita*	Soybean	Mississippi	MK102778	MK102787	
53	TK15	*M. incognita*	Soybean	Mississippi	MK102778	MK102787	
54	TK16	*M. incognita*	Soybean	Mississippi	MK102778	MK102787	MK102799
55	TK17	*M. incognita*	Soybean	Mississippi		MK102787	
56	TK18	*M. incognita*	Corn	Mississippi		MK102787	
57	TK19	*M. incognita*	Soybean	Mississippi	MK102778	MK102787	
58	TK20	*M. incognita*	Soybean	Mississippi	MK102778	MK102787	
59	TK21	*M. incognita*	Soybean	Mississippi	MK102776	MK102787	MK102799
60	TK22	*M. incognita*	Soybean	Lonoke		MK102787	MK102799
61	TK23	*M. incognita*	Soybean	Lonoke	MK102776	MK102787	
62	TK24	*M. incognita*	Soybean	Lonoke	MK102776	MK102787	MK102799
63	TK25	*M. incognita*	Soybean	Lonoke	MK102777	MK102787	
64	TK26	*M. incognita*	Soybean	Pulaski	MK102776	MK102786	
65	TK27	*M. incognita*	Corn	Randolph	MK102776	MK102787	MK102799
66	TK28	*M. incognita*	Soybean	Randolph	MK102778	MK102787	
67	TK29	*M. incognita*	Soybean	Chicot	MK102777	MK102787	
68	TK30	*M. incognita*	Soybean	Chicot	MK102779	MK102787	MK102799
69	TK31	*M. incognita*	Soybean	Chicot	MK102776	MK102787	MK102799
70	TK32	*M. incognita*	Soybean	Chicot	MK102778	MK102787	
71	TK33	*M. incognita*	Soybean	Mississippi	MK102779	MK102787	MK102799
72	TK34	*M. incognita*	Grain Sorghum	Mississippi	MK102779	MK102787	
73	TK35	*M. incognita*	Grain Sorghum	Mississippi		MK102787	MK102799
74	TK36	*M. incognita*	Grain Sorghum	Mississippi	MK102776	MK102787	MK102799
75	TK37	*M. incognita*	Grain Sorghum	Mississippi	MK102779	MK102787	
76	TK38	*M. incognita*	Grain Sorghum	Mississippi	MK102779	MK102787	MK102799
77	TK39	*M. incognita*	Soybean	Mississippi		MK102787	MK102799
78	TK40	*M. incognita*	Soybean	Mississippi		MK102787	
79	TK41	*M. incognita*	Soybean	Mississippi		MK102787	MK102799
80	TK42	*M. incognita*	Grain Sorghum	Mississippi			
81	TK43	*M. incognita*	Soybean	Mississippi		MK102787	MK102799
82	TK44	*M. incognita*	Soybean	Mississippi	MK102779	MK102787	MK102799
83	TK45	*M. incognita*	Soybean	Mississippi		MK102787	MK102799
84	TK46	*M. incognita*	Soybean	Mississippi	MK102778	MK102787	MK102799
85	TK47	*M. incognita*	Soybean	Mississippi	MK102776	MK102787	MK102799
86	TK48	*M. incognita*	Soybean	Mississippi		MK102787	MK102799
87	TK49	*M. incognita*	Soybean	Mississippi	MK102776	MK102787	MK102799
88	TK50	*M. incognita*	Soybean	Mississippi	MK102778	MK102787	MK102799
89	TK51	*M. incognita*	Soybean	Mississippi		MK102787	MK102799
90	TK52	*M. incognita*	Soybean	Mississippi		MK102787	MK102799
91	TK53	*M. incognita*	Soybean	Mississippi		MK102787	MK102799
92	TK54	*M. incognita*	Soybean	Mississippi	MK102779	MK102787	MK102799
93	TK55	*M. incognita*	Soybean	Mississippi	MK102779	MK102787	MK102799
94	TK56	*M. incognita*	Soybean	Mississippi	MK102779	MK102787	MK102799
95	TK57	*M. incognita*	Soybean	Mississippi	MK102779	MK102787	MK102799
96	TK58	*M. incognita*	Soybean	Mississippi	MK102779	MK102787	MK102799
97	TK59	*M. incognita*	Soybean	Mississippi	MK102779	MK102787	MK102799
98	TK60	*M. incognita*	Soybean	Mississippi		MK102787	MK102799
99	TK61	*M. incognita*	Soybean	Mississippi	MK102776	MK102787	MK102799
100	TK62	*M. incognita*	Soybean	Mississippi		MK102787	MK102799
101	TK63	*M. incognita*	Soybean	Drew	MK102779	MK102787	MK102799
102	TK64	*M. incognita*	Soybean	Drew	MK102779	MK102787	MK102799
103	TK65	*M. incognita*	Soybean	Drew		MK102787	MK102799
104	TK66	*M. incognita*	Corn	Drew		MK102787	MK102799
105	TK67	*M. incognita*	Corn	Drew	MK102778	MK102787	MK102799
106	TK68	*M. incognita*	Soybean	Mississippi	MK102778	MK102787	MK102799
107	TK69	*M. incognita*	Soybean	Prairie	MK102779	MK102787	MK102799
108	TK70	*M. incognita*	Soybean	Mississippi		MK102787	MK102799
109	TK71	*M. incognita*	Soybean	Mississippi		MK102787	MK102799
110	TK72	*M. incognita*	Soybean	Craighead	MK102779	MK102787	MK102799
111	TK73	*M. incognita*	Soybean	Mississippi		MK102787	MK102799
112	TK74	*M. incognita*	Soybean	Craighead	MK102779	MK102787	MK102799
113	TK75	*M. incognita*	Soybean	Craighead	MK102776		MK102799
114	TK76	*M. incognita*	Soybean	Craighead	MK102778	MK102787	MK102799
115	TK77	*M. incognita*	Soybean	Mississippi		MK102787	MK102799
116	TK78	*M. incognita*	Soybean	Mississippi	MK102778	MK102787	MK102799
117	TK79	*M. incognita*	Soybean	Mississippi		MK102787	MK102799
118	TK80	*M. incognita*	Soybean	Mississippi		MK102787	MK102799
119	TK81	*M. incognita*	Soybean	Mississippi		MK102787	MK102799
120	TK82	*M. incognita*	Soybean	Mississippi	MK102778	MK102787	
121	TK83	*M. incognita*	Soybean	Mississippi	MK102779	MK102787	MK102799
122	TK84	*M. incognita*	Soybean	Mississippi		MK102787	MK102799
123	TK85	*M. incognita*	Soybean	Mississippi	MK102779	MK102787	MK102799
124	TK86	*M. incognita*	Soybean	Mississippi	MK102778	MK102787	MK102799
125	TK87	*M. incognita*	Soybean	Mississippi		MK102787	MK102799
126	TK88	*M. incognita*	Soybean	Mississippi		MK102787	MK102799
127	TK89	*M. incognita*	Soybean	Mississippi	MK102778	MK102787	MK102799
128	TK90	*M. incognita*	Soybean	Mississippi			MK102799
129	TK91	*M. incognita*	Soybean	Mississippi	MK102778	MK102787	MK102799
130	TK92	*M. incognita*	Soybean	Mississippi		MK102787	MK102799
131	TK93	*M. incognita*	Soybean	Mississippi	MK102778	MK102787	MK102799
132	TK94	*M. incognita*	Soybean	Mississippi	MK102776	MK102787	MK102799
133	TK95	*M. incognita*	Soybean	Mississippi	MK102778	MK102787	
134	TK96	*M. incognita*	Soybean	Mississippi		MK102787	MK102799
135	TK97	*M. incognita*	Soybean	Mississippi	MK102776	MK102787	MK102799
136	TK98	*M. incognita*	Soybean	Craighead	MK102776	MK102787	MK102799
137	TK99	*M. incognita*	Soybean	Mississippi	MK102778		
138	TK100	*M. incognita*	Corn	Desha	MK102779	MK102787	MK102802
139	TK101	*M. incognita*	Soybean	Craighead	MK102779	MK102787	MK102799
140	TK102	*M. incognita*	Soybean	Craighead	MK102776	MK102787	
141	TK103	*M. incognita*	Soybean	Craighead	MK102778	MK102787	
142	TK104	*M. incognita*	Soybean	Craighead	MK102776	MK102791	MK102799
143	TK105	*M. incognita*	Soybean	Lonoke	MK102778	MK102787	MK102799
144	TK106	*M. incognita*	Soybean	Lonoke	MK102776	MK102787	MK102799
145	TK107	*M. incognita*	Soybean	Cross		MK102787	
146	TK108	*M. incognita*	Soybean	Cross	MK102778	MK102787	MK102799
147	TK109	*M. incognita*	Soybean	Jackson		MK102787	MK102799
148	TK110	*M. incognita*	Soybean	Jackson		MK102787	MK102799
149	TK111	*M. incognita*	Soybean	Jackson	MK102778	MK102787	
150	TK112	*M. incognita*	Soybean	Jackson		MK102787	
151	TK113	*M. incognita*	Soybean	Pope	MK102778	MK102787	MK102799
152	TK114	*M. incognita*	Soybean	Woodruff	MK102778	MK102787	MK102801
153	TK115	*M. incognita*	Soybean	Jefferson	MK102778	MK102791	MK102799
154	TK116	*M. incognita*	Soybean	Woodruff	MK102778	MK102787	MK102801
155	TK117	*M. incognita*	Soybean	Craighead	MK102778	MK102787	MK102799
156	TK118	*M. incognita*	Soybean	Lafayette		MK102787	MK102799
157	TK119	*M. incognita*	Corn	Lafayette	MK102776	MK102787	
158	TK120	*M. incognita*	Corn	Lafayette	MK102778	MK102787	MK102799
159	TK121	*M. incognita*	Corn	Lafayette	MK102778	MK102787	MK102799
160	TK122	*M. incognita*	Soybean	Desha		MK102787	MK102799
161	TK123	*M. incognita*	Soybean	Desha	MK102778	MK102787	MK102799
162	TK124	*M. incognita*	Corn	Desha	MK102777	MK102787	
163	TK125	*M. incognita*	Soybean	Desha		MK102790	MK102799
164	TK126	*M. incognita*	Soybean	Desha	MK102779	MK102787	MK102799
165	TK127	*M. incognita*	Soybean	Lincoln	MK102778	MK102787	MK102799
166	TK128	*M. incognita*	Soybean	Lincoln	MK102778	MK102787	MK102799
167	TK129	*M. incognita*	Soybean	Mississippi	MK102778	MK102787	MK102799
168	TK130	*M. incognita*	Soybean	Mississippi	MK102778	MK102787	MK102799
169	TK131	*M. incognita*	Soybean	Mississippi	MK102778	MK102787	MK102799
170	TK132	*M. incognita*	Soybean	Mississippi		MK102787	MK102799
171	TK133	*M. incognita*	Soybean	Mississippi	MK102778	MK102787	MK102799
172	TK134	*M. incognita*	Soybean	Mississippi	MK102779	MK102787	MK102799
173	TK135	*M. incognita*	Soybean	Mississippi	MK102778	MK102787	MK102799
174	TK136	*M. incognita*	Soybean	Mississippi	MK102778	MK102787	MK102799
175	TK137	*M. incognita*	Soybean	Mississippi	MK102778	MK102787	MK102799
176	TK138	*M. incognita*	Soybean	Desha	MK102778	MK102787	MK102799
177	TK139	*M. incognita*	Soybean	Desha	MK102778	MK102787	MK102799
178	TK140	*M. incognita*	Soybean	Desha	MK102778	MK102787	MK102799
179	TK141	*M. incognita*	Soybean	Mississippi	MK102778	MK102787	MK102799
180	TK142	*M. incognita*	Soybean	Mississippi	MK102778	MK102787	MK102799
181	TK143	*M. incognita*	Soybean	Mississippi	MK102778	MK102787	MK102799
182	TK144	*M. incognita*	Soybean	Mississippi	MK102778	MK102787	MK102799
183	TK145	*M. incognita*	Soybean	Mississippi	MK102778	MK102787	MK102799
184	TK146	*M. incognita*	Soybean	Mississippi	MK102778	MK102787	MK102799
185	TK147	*M. incognita*	Soybean	Mississippi	MK102778	MK102787	MK102799
186	TK148	*M. incognita*	Soybean	Mississippi	MK102778	MK102790	MK102799
187	TK149	*M. incognita*	Soybean	Mississippi	MK102778	MK102787	MK102799
188	TK150	*M. incognita*	Soybean	Mississippi	MK102778	MK102787	MK102799
189	TK151	*M. incognita*	Soybean	Mississippi	MK102776	MK102787	MK102799
190	TK152	*M. incognita*	Soybean	Mississippi	MK102776	MK102787	MK102799
191	TK153	*M. incognita*	Soybean	Mississippi	MK102778	MK102787	MK102799
192	TK154	*M. incognita*	Soybean	Mississippi		MK102787	MK102799
193	TK155	*M. incognita*	Soybean	Mississippi	MK102778	MK102787	MK102799
194	TK156	*M. incognita*	Soybean	Mississippi	MK102778	MK102787	MK102799
195	TK157	*M. incognita*	Soybean	Desha	MK102776	MK102787	MK102801
196	TK158	*M. incognita*	Soybean	Crittenden	MK102779	MK102787	MK102801
197	TK159	*M. incognita*	Soybean	Crittenden		MK102787	MK102799
198	TK160	*M. incognita*	Soybean	Crittenden	MK102778	MK102787	MK102799
199	TK161	*M. incognita*	Soybean	Crittenden	MK102776	MK102787	MK102799
200	TK162	*M. incognita*	Soybean	Greene	MK102777	MK102787	MK102798
201	TK163	*M. incognita*	Soybean	Clay	MK102778	MK102787	MK102799
202	TK164	*M. incognita*	Soybean	Desha	MK102778	MK102787	MK102801
203	TK165	*M. incognita*	Soybean	Clay	MK102778	MK102789	MK102799
204	TK166	*M. incognita*	Soybean	Clay	MK102778	MK102787	MK102799
205	TK167	*M. incognita*	Soybean	Conway	MK102778	MK102787	MK102799
206	TK168	*M. incognita*	Soybean	Lawrence	MK102778	MK102787	MK102799
207	TK169	*M. incognita*	Soybean	Conway	MK102778	MK102787	MK102799
208	TK170	*M. incognita*	Soybean	Lawrence	MK102778	MK102787	MK102799
209	TK171	*M. incognita*	Soybean	Desha	MK102778	MK102787	MK102799
210	TK172	*M. incognita*	Soybean	Craighead	MK102778	MK102788	MK102799
211	TK173	*M. incognita*	Soybean	Lawrence	MK102778	MK102787	MK102799
212	TK174	*M. incognita*	Soybean	Lawrence	MK102778	MK102787	MK102799
213	TK175	*M. incognita*	Soybean	Lawrence	MK102778	MK102787	
214	TK176	*M. incognita*	Soybean	Mississippi	MK102778	MK102787	MK102799
215	TK177	*M. incognita*	Soybean	Mississippi	MK102778	MK102787	MK102799
216	TK178	*M. incognita*	Soybean	Desha	MK102778	MK102787	MK102799
217	TK179	*M. incognita*	Soybean	Desha	MK102778	MK102787	MK102799
218	TK180	*M. incognita*	Soybean	Desha	MK102776	MK102787	MK102799
219	TK181	*M. incognita*	Soybean	Desha	MK102778	MK102787	MK102799
220	TK182	*M. incognita*	Soybean	Desha	MK102778	MK102787	MK102799
221	TK183	*M. incognita*	Soybean	Desha	MK102778	MK102787	MK102799
222	TK184	*M. incognita*	Soybean	Woodruff	MK102778	MK102787	MK102799
223	TK185	*M. incognita*	Soybean	Miller	MK102778	MK102789	MK102799
224	TK186	*M. incognita*	Soybean	Miller	MK102778	MK102787	MK102799
225	TK187	*M. incognita*	Soybean	Miller	MK102778	MK102787	MK102799
226	TK188	*M. incognita*	Soybean	Miller	MK102778	MK102787	MK102799
227	TK189	*M. incognita*	Soybean	Miller	MK102778	MK102787	MK102799
228	TK190	*M. incognita*	Soybean	Miller		MK102787	MK102799
229	TK191	*M. incognita*	Soybean	Miller	MK102778	MK102787	MK102799
230	TK192	*M. incognita*	Soybean	Miller	MK102778	MK102787	MK102799
231	TK193	*M. incognita*	Soybean	Desha	MK102778	MK102787	MK102799
232	TK194	*M. incognita*	Soybean	Desha	MK102779	MK102787	MK102799
233	TK195	*M. incognita*	Soybean	Desha	MK102778	MK102787	
234	TK196	*M. incognita*	Soybean	Desha	MK102776	MK102787	MK102799
235	TK197	*M. incognita*	Soybean	Ashley	MK102779	MK102787	MK102799
236	TK198	*M. incognita*	Soybean	Ashley		MK102787	
237	TK199	*M. incognita*	Soybean	Desha	MK102776	MK102787	MK102799
238	TK200	*M. incognita*	Soybean	Randolph	MK102778	MK102789	MK102799
239	TK201	*M. haplanaria*	Soybean	Logan	MK102771	MK102784	MK102793
240	TK202	*M. incognita*	Soybean	Randolph	MK102778	MK102787	MK102799
241	TK203	*M. incognita*	Soybean	Logan	MK102779	MK102787	MK102799
242	TK204	*M. incognita*	Soybean	Johnson	MK102778	MK102787	MK102798
243	TK205	*M. incognita*	Soybean	Clay	MK102776	MK102787	MK102799
244	TK206	*M. incognita*	Soybean	Lincoln	MK102776	MK102787	MK102799

**Table 2:** Primers used for polymerase chain reaction and DNA sequencing.

Primer	Gene	Sequence (5′ to 3′)	Reference
Me18S17F	18S	GAGAAACCGCGAACGGCTCA	^36^
Me18S500F	18S	GCAAGTCTGGTGCCAGCAGC	^36^
Me18S740R	18S	TCCATGCACGATCATTCAAGCG	^36^
Me18S840F	18S	ATTTGTATGGTCCCGTGAGAGG	^36^
Me18S940R	18S	TGATCGCCTTCGAACCTCTG	^36^
Me18S1120F	18S	ACCACCAGGAGTGGAGCC	^36^
Me18S1120R	18S	GGCTCCACTCCTGGTGGT	^36^
Me18S1220R	18S	ATGCACCACCATCCACTGAATC	^36^
Me18S1710R	18S	GCCCGGTTCAAGCCACTG	^36^
Me18S1740R	18S	GCAGGTTCACCTACAGCTACCT	^36^
RKITSF2	ITS	GTAGGTGAACCTGCTGCTG	^36^
MeITS2R	ITS	ATGCTTAAGTTCAGCGGGTG	^36^
RK28SF	28S D2/D3	CGGATAGAGTCGGCGTATC	^36^
RK28SR	28S D2/D3	GATGGTTCGATTAGTCTTTCGCC	^36^
RK28SUR	28S D2/D3	CCCTATACCCAAGTCAGACGAT	^36^
C2F3	CoxII-IGS	GGTCAATGTTCAGAAATTTGTGG	^71^
ITSUniF	18S-ITS	GTGCATGGCCGTTCTTAGTT	This study
Nxy22	18S-ITS	TTCACTGCGTTCTTCATCGATC	This study
MeloCOIIR	CoxII-IGS	CGATCTTTATCAGGATGAGCACC	This study
Melo16SR	CoxII-IGS	CCTTTGACCAATCACGCTAAAAGTGC	This study
Inc–K14-F	SCAR	CCCGCTACACCCTCAACTTC	^69^
Inc–K14-R	SCAR	GGGATGTGTAAATGCTCCTG	^69^
Finc	SCAR	CTCTGCCCAATGAGCTGTCC	^22^
Rinc	SCAR	CTCTGCCCTCACATTAAG	^22^
Fjav	SCAR	GGTGCGCGATTGAACTGAGC	^22^
Rjav	SCAR	CAGGCCCTTCAGTGGAACTATAC	^22^
Far	SCAR	TCGGCGATAGAGGTAAATGAC	^22^
Rar	SCAR	TCGGCGATAGACACTACAACT	^22^
MH0F	SCAR	CAGGCCCTTCCAGCTAAAGA	^70^
MH1R	SCAR	CTTCGTTGGGGAACTGAAGA	^70^

### DNA sequencing

The rDNA 18S-ITS-5.8S (182 sequences), 28S D2/D3 (226 sequences) and CoxII-IGS (197 sequences) were deposited in GenBank and their GenBank accession numbers are presented in Table 1. Although attempts were made to perform DNA sequencing on all three genes for each sample, not all PCR or DNA sequencing was successful. However, at least one gene was sequenced from all RKN populations except for one population (TK42). One hundred forty-two samples (58.2%) have all three genes sequenced. Many of the sequences from different populations are identical, thus their sequences were assigned the same accession number. Minor DNA sequence variations within the same species were observed in each gene among some populations.

DNA sequences of MK102775 (1,980 bp), MK102776 (2,296 bp), MK102777 (1,227 bp), MK102778 (968 bp) and MK102779 (1,815 bp) are different regions of 18S-ITS-5.8S and have more than 99% identity with many sequences of *M. incognita*, *M. javanica* and *M. arenaria* from GenBank. MK102771 (2,180 bp), MK102772 (2,216 bp) and MK102773 (2,180 bp) matched with two sequences of *M. haplanaria* (AY919178, 637 bp and AY757867, 637 bp) with 100% identity in aligned region. These three sequences are 98–99% identical to many tropical species sequences including *M. incognita*, *M. javanica* and *M. arenaria* from GenBank. The 2,215-bp DNA sequence of 18S-ITS-5.8S (MK102780) is 99–100% identical to DNA sequences of *M. hapla* from the GenBank (KP901065, KJ636268, AY268119, AY593892, EU669941, EU669942, AY942628, MH011983, EU669943 and KJ636267). DNA sequences of MK102774 (2,015 bp) and MK102781 (790 bp) are 100% identical to *M. marylandi* (KP901041) and 99% identical to *M. marylandi* (KP901049 and KP901043).

The DNA sequence of 28S D2/D3 (MK102787, 1,006 bp) of *M. incognita* is fairly conserved; no sequence variation was observed among most Arkansas populations. It has minor nucleotide differences with other Arkansas sequences of *M. incognita* (MK102786, 1,006 bp, MK102788, 643 bp, MK102789, 641 bp, MK102790, 643 bp, and MK102791, 643 bp). Blast search of these sequences revealed 97–100% identity with many tropical species sequences including *M. incognita*, *M. javanica* and *M. arenaria* from GenBank (KP901082, KP901083, KP901078, etc.). The DNA sequences of 28S D2/D3 (MK102784, 1,003 bp and MK102785, 935 bp) on *M. haplanaria* are 95–96% identical to many tropical species sequences including *M. incognita*, *M. javanica* and *M. arenaria* from GenBank (KP901082, KP901083, KP901078, etc.). No 28S DNA sequence of *M. haplanaria* from GenBank is available to compare with the study populations. The 1,042-bp DNA sequence (MK102780) of *M. hapla* is 99–100% identical to DNA sequences of *M. hapla* from GenBank (GQ130139, KU180679, KP306534, KP306532, KU587712, KP901086, DQ145641, KJ598136 and KJ755183). The 678-bp DNA sequence (MK102782) of *M. marylandi* is close to many sequences of *M. marylandi* (KP901066 etc.) with 99–100% identity. The 667-bp DNA sequence of *M. partityla* (MK102783) is 94% identical to *M. ethiopica* (KY882483), *M. hispanica* (EU443606) and *M. luci* (LN626951). No 28S DNA sequence of *M. partityla* from GenBank is available to compare with the study population.

**Figure 9:**
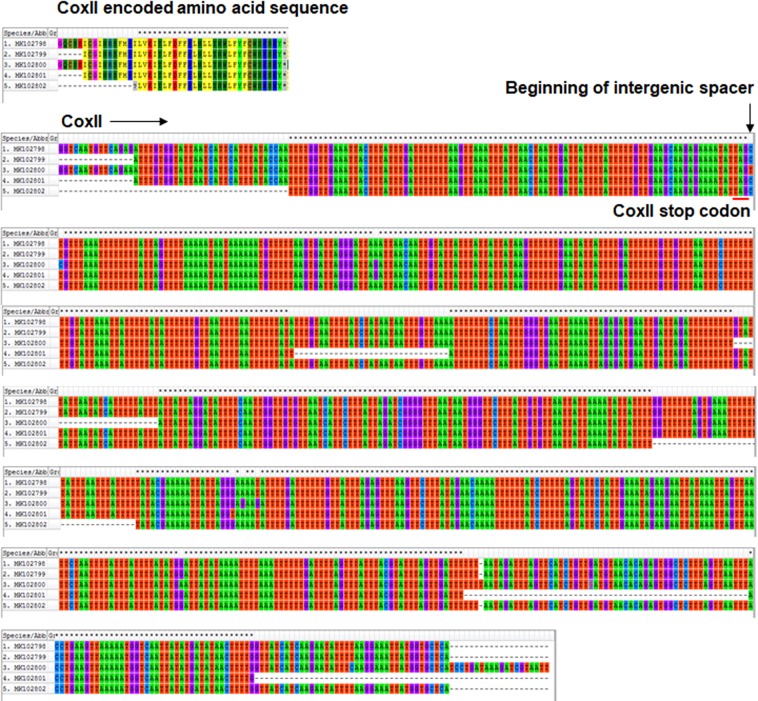
Multiple alignment of CoxII-IGS gene in *Meloidogyne incognita* collected from Arkansas.

The DNA sequences of mitochondrial DNA CoxII-IGS of *M. incognita* (MK102798, 912 bp, MK102799, 879 bp, MK102800, 909 bp, MK102801, 771 bp, MK102802, 831 bp) are comprised of 138-bp CoxII and the rest IGS. The CoxII sequences are highly conserved and identical which encode a polypeptide GQCSEICGINHSFMPILVEITLFDFFKLNLLTNWLFYFCWSKSKY. However, there are five types of IGS sequences that showed four significant gaps, six mutations and one insertion/deletion as shown in Fig. 9. Blast search of these sequences revealed 99–100% identity to many tropical species sequences including *M. incognita*, *M. javanica* and *M. arenaria* from GenBank (MH152335, MF043913, LN864824, etc.). The DNA sequences of CoxII-IGS (MK102793, 660 bp, MK102794, 541 bp and MK102795, 541 bp) on *M. haplanaria* are 99% identical to sequences of *M. haplanaria* (KT783539, KM881682, AY757905 and AY757906). The 470-bp DNA sequence (MK102792) of *M. hapla* is 99% identical to DNA sequences of *M. hapla* from the GenBank (KJ598134, AY757887, AY757888, AY757899, KP681265, KM881684 and KF993633). The 533-bp DNA sequence (MK102797) of *M. marylandi* is identical to sequence of *M. marylandi* (JN241918) and a few bp differences with other sequences of *M. marylandi* (JN241917, KM881683 and KC473862). The 511-bp DNA sequence of *M. partityla* (MK102796) is 99% identical to *M. partityla* (AY672412, AY757908, AY672413 and KM881686).

### Molecular phylogenetic relationships

**Figure 10:**
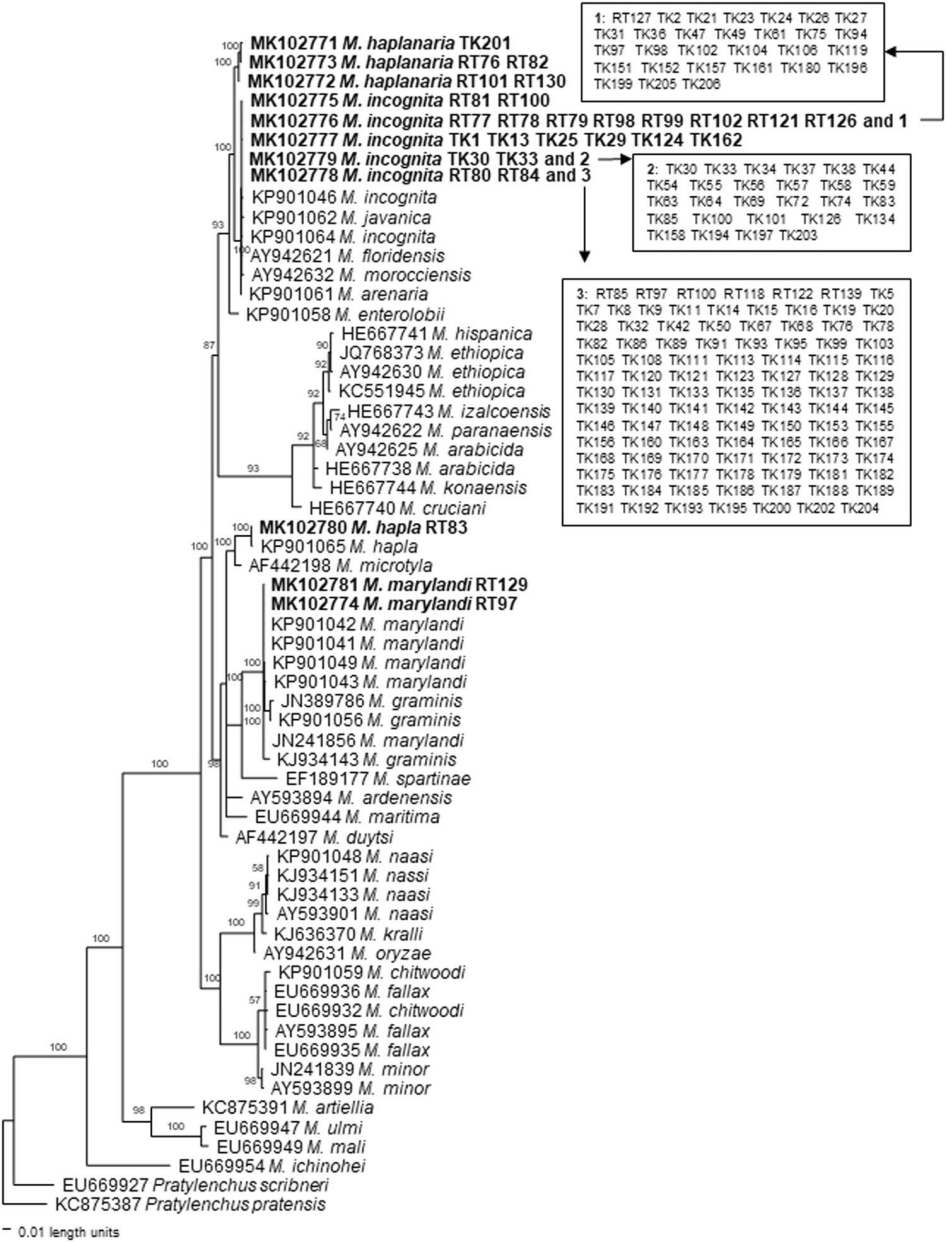
Bayesian consensus tree inferred from rDNA 18S-ITS-5.8S under GTR + I + G model (-lnL = 13647.8496; AIC = 27315.6992; freqA = 0.2616; freqC = 0.2077; freqG = 0.2494; freqT = 0.2813; R(a) = 1.2697; R(b) = 2.0864; R(c) = 1.6566; R(d) = 0.6843; R(e) = 3.1581; R(f) = 1; Pinva = 0.3599; Shape = 0.3398). Posterior probability values exceeding 50% are given on appropriate clades.

A phylogenetic tree based on the rDNA 18S-ITS-5.8S is presented in Fig. 10 with two *Pratylenchus* species as outgroup taxa. This tree placed the study populations in three distinct groups. *Meloidogyne incognita* populations are in a clade with other tropical RKN species including *M. incognita, M. arenaria*, *M. javanica*, *M. floridensis* and *M. morocciensis* with 100% support. *Meloidogyne haplanaria* is sister to this clade with 100% support. *Meloidogyne enterolobii* is basal to this clade with 93% support. *Meloidogyne marylandi* and *M. graminis* are very closely related and are in a clade with *M. spartinae* with 100% support*. Meloidogyne hapla* is sister to *M. microtyla* with 100% support. *Meloidogyne hapla* and *M. marylandi* are in a monophyletic group with 100% support. Unfortunately, *M. partityla* from this study was not sequenced successfully.

**Figure 11:**
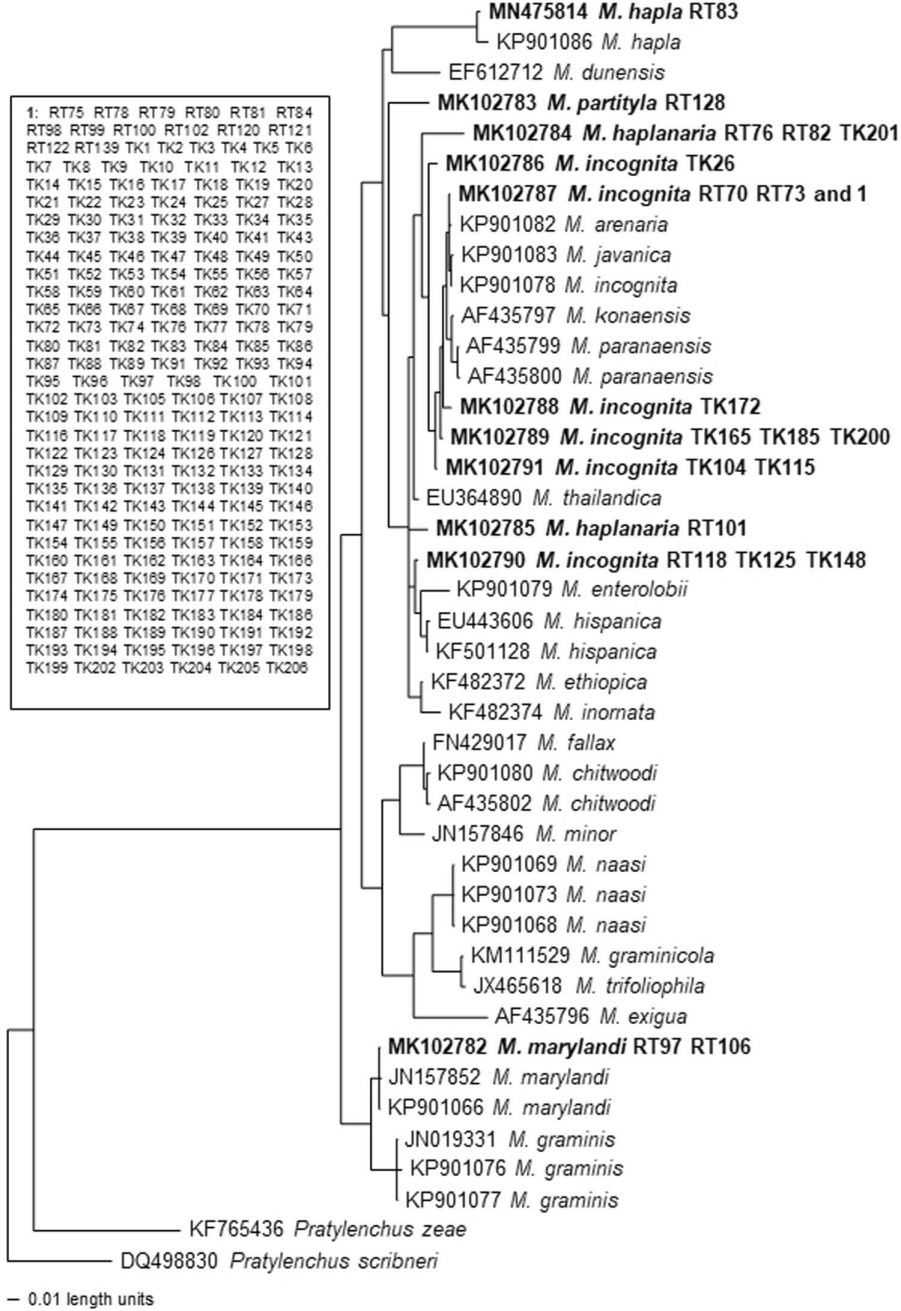
Bayesian consensus tree inferred from rDNA 28S D2/D3 under TVM + I + G model (-lnL = 5664.7959; AIC = 11347.5918; freqA = 0.2548; freqC = 0.1889; freqG = 0.2676; freqT = 0.2888; R(a) = 0.6653; R(b) = 3.0047; R(c) = 1.7303; R(d) = 0.3041; R(e) = 3.0047; R(f) = 1; Pinva = 0.2636; Shape = 0.6053). Posterior probability values exceeding 50% are given on appropriate clades.

A phylogenetic tree based on the rDNA 28S D2/D3 sequences is presented in Fig. 11 with two *Pratylenchus* species as outgroup taxa. This tree placed Arkansas RKN in four distinct groups. *Meloidogyne hapla* population RT83 (MN475814) is in a clade with *M. hapla* (KP901086). This clade is in a monophyletic clade with *M. dunensis* (EF612712) with 84% support. *Meloidogyne incognita* (MK102786-MK102791) and *M. haplanaria* (MK102784 and MK102785) are in a monophyletic clade with *M. arenaria*, *M. javanica*, *M. incognita, M. konaensis, M. paranaensis, M. thailandica, M. enteroloii, M. hispanica, M. ethiopica* and *M. inornata* with 100% support*. Meloidogyne partityla* is sister to this clade with 82% support. *Meloidogyne marylandi* (MK102782) is in a clade with *M. marylandi* (JN157852 and KP901066) and *M. graminis* (JN019331, KP901076 and KP901077) with 99% support.

**Figure 12:**
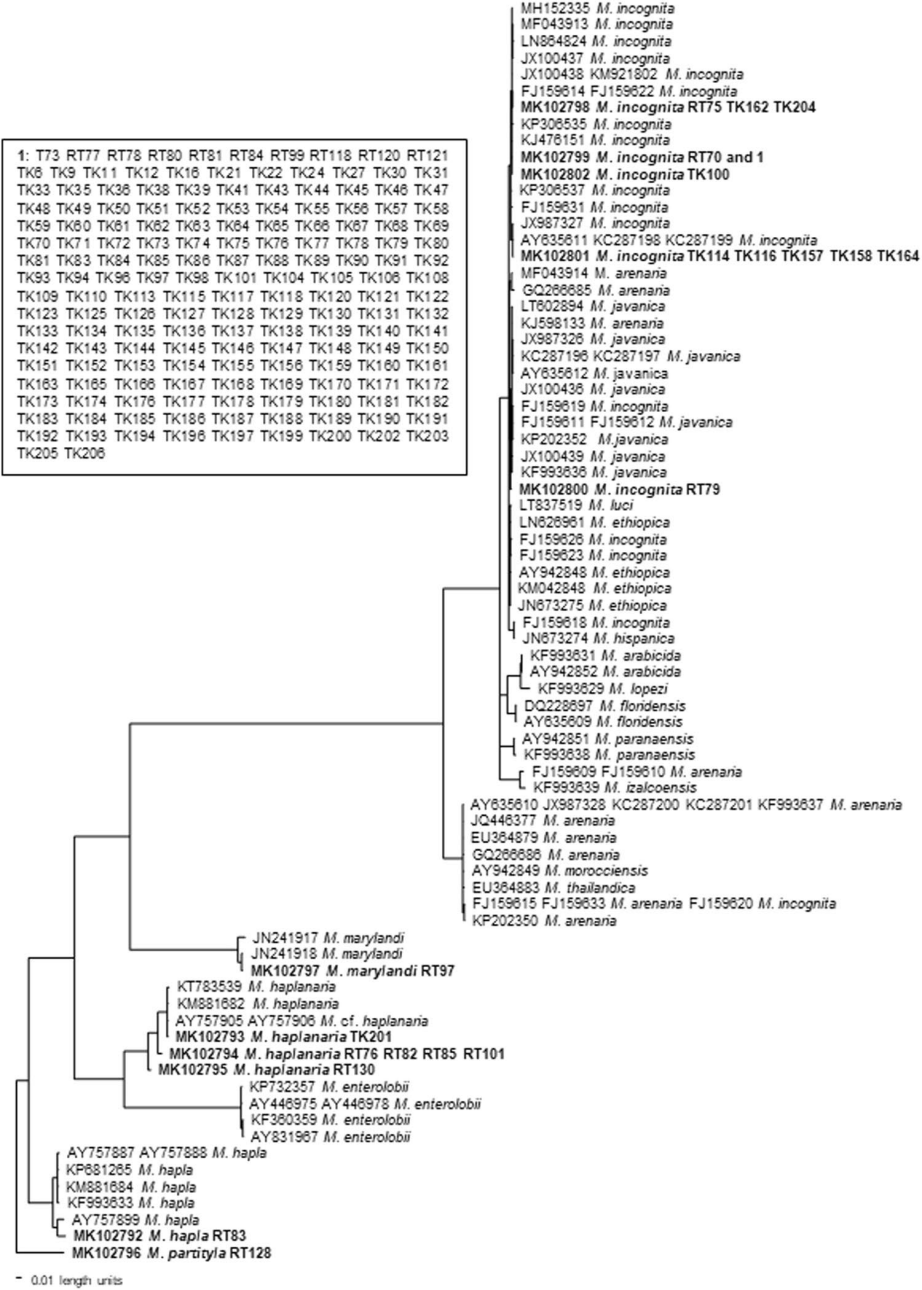
Bayesian consensus tree inferred from mitochondrial DNA CoxII-IGS under TVM + G model (-lnL = 4936.4829; AIC = 9888.9658; freqA = 0.3513; freqC = 0.0315; freqG = 0.1032; freqT = 0.5139; R(a) = 2.3466; R(b) = 4.1635; R(c) = 1.2778; R(d) = 4.0003; R(e) = 4.1635; R(f) = 1; Pinva = 0; Shape = 0.7173). Posterior probability values exceeding 50% are given on appropriate clades.

A phylogenetic tree based on the mitochondrial DNA CoxII-IGS sequences is presented in Fig. 12 rooted with *M. partityla* (MK102796) based on the multiple sequence alignment whose sequence is most distinct from the other sequences. No outgroup species was included in the analysis because of the large sequence divergency. This tree placed Arkansas RKN in five distinct groups. *Meloidogyne partityla* (MK102796) is at the basal position. *Meloidogyne hapla* population RT83 (MK102792) is in a clade with other *M. hapla* (AY757887, AY757888, KP681265, KM881684, KF993633 and AY757899). *Meloidogyne haplanaria* (MK102793-MK102795) is in a clade with other *M. haplanaria* (KT783539, KM881682, AY757905 and AY757906). This clade is sister to *M. enterolobii* with 100% support. *Meloidogyne marylandi* (MK102797) is in a clade with two other *M. marylandi* (JN241917 and JN241918). *Meloidogyne incognita* (MK102798- MK102802) is in a monophyletic clade with *M. incognita, M. arenaria*, *M. javanica*, *M. luci, M. ethiopica, M. arabicida, M. lopezi, M. paranaensis*, and *M. izalcoensis* with 98% support. This clade is sister to *M. arenaria*, *M. morocciensis*, *M. thailandica* and *M. incognita* with 100% support.

### PCR by species-specific primers

**Figure 13:**
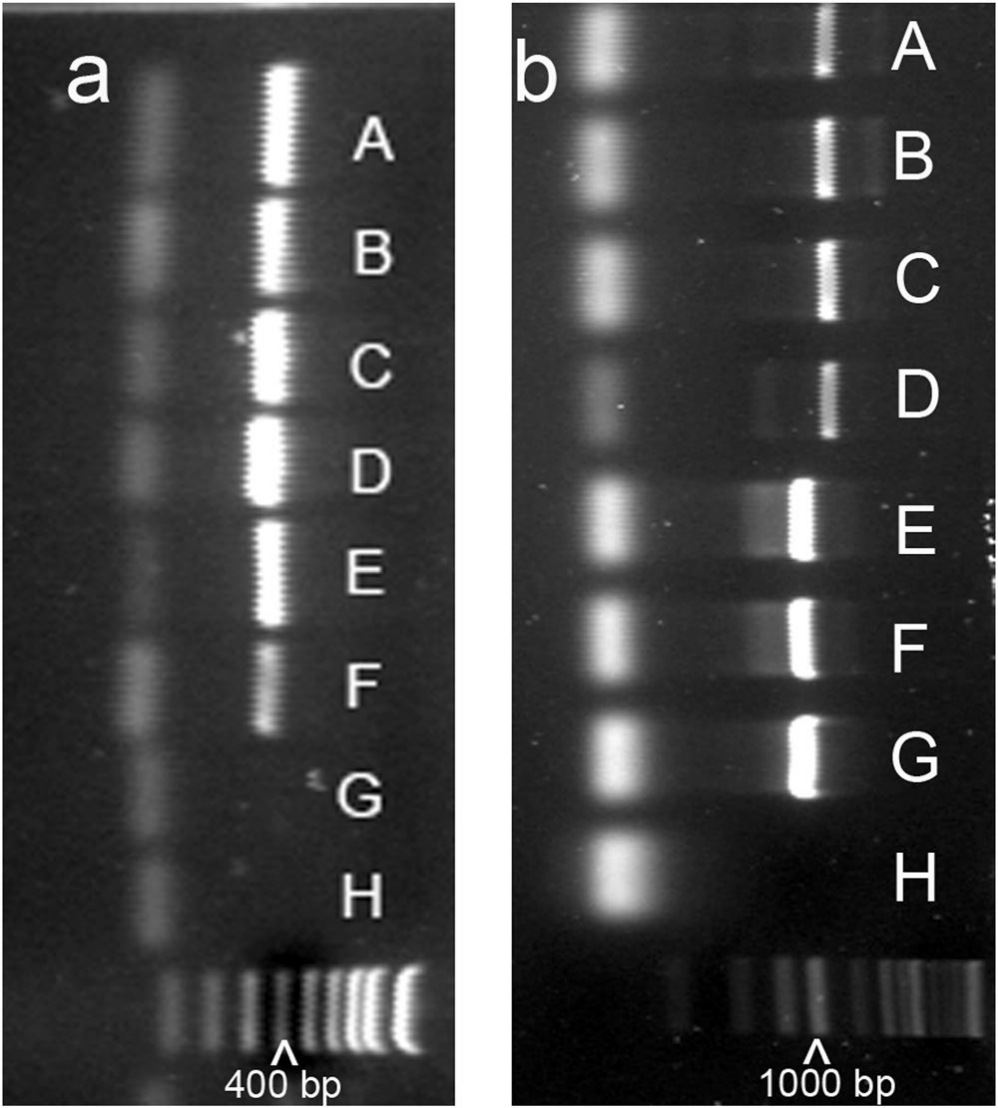
Photographs of an example of agarose gel electrophoresis of root-knot nematode (*Meloidogyne* spp.) from Arkansas by species-specific primers. (**a**) Primers Inc-K14-F/Inc-K14-R, *M. incognita*-specific. Lane A: TK3; B: TK42; C: TK156; D: TK196; E: TK206; F: RT131; G: RT128; H: Water negative control; 100 bp low scale DNA ladder. (**b**) A–D: primers Finc/Rinc, *M. incognita*-specific; E–H: primers MH0F/MH1R, *M. hapla*-specific. Lane A: TK3; B: TK42; C: TK190; D: RT137; E: RT83-female 1; F: RT83-female 2; G: VW9, *M. hapla*-positive control; H: Water negative control; 1 kb DNA ladder.

The species identification of *M. incognita* was confirmed using PCR by *M. incognita*-specific SCAR primers Inc-K14-F/Inc-K14-R which produced a 399-bp DNA fragment (Fig. 13a) or Finc/Rinc which produced a 1200-bp PCR fragment (Fig. 13b). Only one population (RT83) is positive to primers MH0F/MH1R which were *M. hapla*-specific with 960-bp amplicon (Fig. 13b). None of these study samples were positive to primers Fjav/Rjav and Far/Rar which are species-specific to *M. javanica* and *M. arenaria* respectively. One population TK42 failed to get any good DNA sequencing results on three genes, but it is positive for *M. incognita* when using PCR by *M. incognita*-specific SCAR primers (Fig. 13).

## Discussion

This study characterized DNA sequences on ribosomal DNA 18S-ITS-5.8S, 28S D2/D3 and a mitochondrial DNA CoxII-IGS on 244 RKN populations from various hosts, collected from 39 counties in Arkansas. Five species were identified, including *M. incognita*, *M. hapla*, *M. haplanaria*, *M. marylandi* and *M. partityla* through a combined analysis of DNA sequencing and PCR by species-specific primers. The phylogenetic relationships agreed broadly, i.e. sequences analysed were grouped into clades as reasonably expected with no contradictions irrespective of the three loci sequenced. Although DNA sequencing can determine *M. hapla*, *M. haplanaria*, *M. marylandi* and *M. partityla* by any of the three genes, it is impossible to determine *M. incognita* because these genes are too conserved among other closely related RKN as shown in blast search and phylogenetic trees. PCR by species-specific primers is needed for the identification of *M. incognita*. Unlike earlier surveys of the state, *M. arenaria*, *M. javanica* and *M. graminis* were not detected from any of the samples. One RKN population with the second-stage juveniles having very short tails was found in a sample collected at the Lon Mann Cotton Research Station near Brinkley, Arkansas. This sample was found below an oak tree in a mixture of grasses and dicot weeds. Several attempts to find females failed and no DNA study was ever performed. There were some RKN samples forwarded to the second author by the Arkansas Nematode Assay Service and by the Arkansas Plant Health Clinic that contained soil with little or no roots, thus only the second-stage juveniles were available. These second-stage juveniles were reared in a greenhouse using tomato and bermudagrass as possible hosts. While some success in producing a population of RKN resulted, most testing resulted in failure. This failure was disappointing in that two samples identified with the second-stage juveniles appeared to be *M*. *arenaria*^17^. Another failure was not establishing a RKN population when finding males along with the second-stage juveniles in grass samples in experimental plots from the main University Experiment Station in Fayetteville.

*Meloidogyne incognita* (Southern RKN) is the most abundant species and was identified in 95% samples. It was the only species found in field crops including soybean and cotton, except for one population of *M. haplanaria* from soybean in Logan County (TK201). This species has worldwide distribution and numerous hosts and is the most damaging species throughout the tropics and warmer regions of the world. *Meloidogyne incognita* is predominantly found in warmer climates, at latitudes between 35°S and 35°N^26^. This study revealed *M. incognita* is the most common and widespread species in field crops in Arkansas.

*Meloidogyne hapla* (Northern RKN) is widely distributed, particularly in temperate regions and the cooler, higher altitude areas of the tropics. Taylor & Buhrer^27^ reported that in the USA, *M. hapla* was most common north of 39°N. It is polyphagous and affects over 550 crops and weeds^28^ including many agricultural and horticultural plants (vegetables, fruits, ornamentals), but few grasses or cereals^28^. From the current and previous study^17^, this species was found from knockout rose, oak, elm and poke weed (*Phytolacca americana*) from three northern counties including Craighead, Logan, and Washington (Fig. 5), but not from any field crops.

*Meloidogyne haplanaria* (Texas peanut RKN) was originally found attacking peanut in Texas^29^ and was also reported from Arkansas^17^ and *Mi*-resistant tomato in Florida^30^. Host range studies revealed that it can parasitize several legumes and crucifer crops^29^ and infect *M. arenaria*-susceptible cultivars of peanut, garden pea and radish^31^. Although watermelon, cotton, corn, tobacco and wheat are nonhosts for *M. haplanaria*, peper, eggplant, soybean and common bean are moderate hosts for this nematode^29,31^. In our study, this species was found on ash, tomato, peanut, willow, elm, Indian hawthorn and soybean from six counties including Baxter, Faulkner, Logan, Saline, Van Buren and Washington (Fig. 6). It’s worthy to note that only one soybean field (TK201) had *M. haplanaria*. This species is distinct by mitochondrial DNA CoxII-IGS, but similar to *M. incognita*, *M. arenaria* and *M. javanica* in ribosomal DNA 18S-ITS and 28S D2/D3.

*Meloidogyne marylandi* (Maryland RKN) was first described by Jepson & Golden^32^ on bermudagrass (*Cynodon dactylon*) in College Park, Maryland, USA. It has been reported from Arkansas^17^, Texas^33^, Florida^34^, Oklahoma^35^, North Carolina, South Carolina^36^, Arizona, California, Nevada, Utah and Hawaii^37^. Outside USA, *M. marylandi* has been found in Japan^38^, Israel^39^, and Costa Rica^40^. From current and previous study^17^, this species was found from grasses from six counties including Craighead, Drew, Hempstead, Logan, Perry and Washington (Fig. 7). Another closely related species, *M. graminis*, is native to USA. It was first described infecting St. Augustine grass (*Stenotaphrum secundatum*) in Winter Haven, Florida, in 1964^41^. This species has been reported on cultivated grasses from Florida to California and Hawaii, as far north as New England, on native grasses in the Konza Prairie in Kansas^42,43^, North Carolina, and South Carolina^36^. The *M. graminis* from grass reported in 1974 by Grisham *et al*.^10^ and in 1982 by Robbins^6^ was believed to be *M. marylandi* which was described much later in 1987^26^. Before *M. marylandi* was described in 1987, no DNA analysis was available and species found from grass in Arkansas was assigned as *M. graminis*. Thus, no *M. graminis* is really confirmed in Arkansas.

*Meloidogyne partityla* (pecan RKN) is a plant pathogenic nematode infecting pecan. It was first described in pecan trees in South Africa by Kleynhans (1986)^44^. It is thought to have been introduced into South Africa by pecan seedlings that came from USA in 1912, 1939 and 1940^44^. Today, this nematode is seen infecting pecan trees in Arizona^45^, Arkansas^46^, Florida^47,48^, Georgia^49^, New Mexico^50^, Oklahoma^45^, South Carolina^51^ and Texas^52^. In addition to pecans, they also infect the California black walnut (*Juglans hindsii*), English walnut (*J. regia*), shagbark hickory (*Carya ovate*), post oak (*Quercus stellate*), water oak (*Quercus nigra*) and laurel oak (*Q. laurifolia*). The health of infested trees continues to decline every year^50^. In this study, only one sample from pecan in Logan County was identified as *M. partityla* (Fig. 8).

*Meloidogyne enterolobii* (Guava RKN) is a recent emerging and highly pathogenic RKN species in the USA. It was originally described from China in 1983^53^ and later reported in Florida in 2004^54^, North Carolina in 2013^55^, Louisiana in 2019^56^ and South Carolina in 2019^57^ attacking field crops, vegetables, ornamental plants, guava tree and weeds. *Meloidogyne enterolobii* is considered as a tropical species; due to its limited distribution and high damage impact, it was added to the European and Mediterranean Plant Protection Organization A2 Alert list^58^ and became a regulated nematode in South Korea, Costa Rica and USA (Florida, Louisiana, Mississippi, North Carolina)^54–60^. Fortunately, *M. enterolobii* was never detected in our survey and thus it is listed as a regulated species to prevent its disperse^61^.

In this study, DNA sequencing and PCR by species-specific primers were employed successfully to characterize and identify RKN from a wide range of plants from 39 counties in Arkansas. The results revealed the presence of five RKN species with *M. incognita* being the most predominant. Their hosts, distribution, DNA sequences of three genes and phylogenetic relationships were investigated. This study provides basic information for future management of these economically important species in Arkansas.

## Methods

### Nematode sample collection

**Figure 14:**
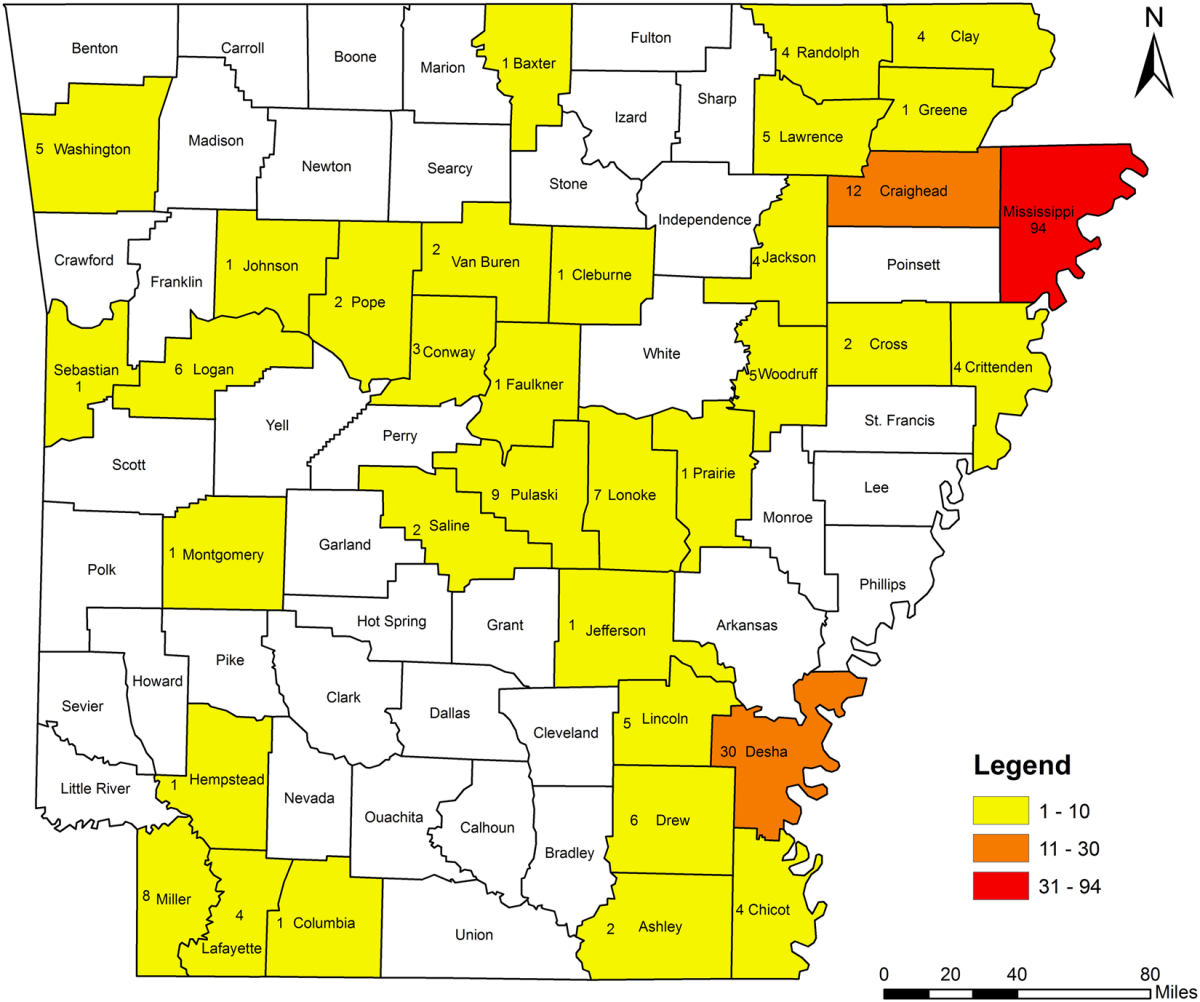
Sampled counties and sample numbers for root-knot nematode survey from Arkansas.

A total of 244 RKN populations from various hosts from 39 counties in Arkansas were sampled in this study from 2014 to 2018 (Table 1) (Fig. 14). These samples were collected during the growing season. No specific permissions were required in sampling for plant-parasitic nematodes and no endangered or protected species were involved. Two hundred and six RKN samples (TK1-TK206) were initially collected from soil samples that were taken by Arkansas Cooperative Extension Service agents as a part of a statewide nematode survey sponsored in part by the Arkansas Soybean Promotion Board. Samples were collected during the period from September 1 – November 1 in 2014–2016 and were from fields that were either in soybean in the year they were sampled, or they were cropped to corn, grain sorghum, or cotton as a rotation crop with soybean. Samples were stored and transported to the Arkansas Nematode Diagnostic Laboratory in Hope, Arkansas in plastic bags inside insulated coolers. Samples were stored no longer than two weeks prior to assay. When RKN was extracted through routine elutriation^62^ and sugar flotation^63^ of a sub-sample, the remaining soil was placed into a 15-cm-diameter clay pot filled with 50:50 mixture of fine builders’ sand and sandy loam topsoil. A single tomato seedling (*Solanum lycopersicon* L var. *lycopersicum*, cv. ‘Rutgers’) at the age of three to four week old from gemination was grown in the soil in a greenhouse. Tomato plants were then removed from the soil and the root systems were washed to remove excess soil at harvest. Root galls on tomato were collected after 60–70 days of inoculation and shipped to Nematode Lab at Agronomic Division in North Carolina Department of Agriculture. Thirty-eight other populations were collected by the second author. Galls or dissected females were shipped to NCDA without rearing nematodes on tomato.

### DNA extraction

RKN females were dissected in water in a 9-cm petri dish under Zeiss Stemi 2000-C microscope (Gottingen, Germany). A single female was pipetted into 10-µl 1X TE buffer (10 mM Tris-Cl, 1 mM EDTA; pH 9.0) on a glass microscope slide (7.5 cm × 2.5 cm). The nematodes were then macerated with a pipette tip into pieces, collected in 50-µl 1X TE buffer and stored at −20 °C. Three DNA replicates per sample were prepared for any samples with females. If only the second-stage juveniles were available, 1–10 juveniles were macerated with a pipette tip into pieces and put in one tube as DNA template in 50-µl 1X TE buffer.

### DNA amplification, cleaning and sequencing

The primers used for ribosomal and mitochondrial DNA PCR and DNA sequencing are shown in Table 2 as previously described^36^. These primers were synthesized by Integrated DNA Technologies, Inc. (Coralville, Iowa, USA). The 25-µl PCR was performed using 12.5-µl 2X Apex *Taq* red master mix DNA polymerase (Genesee Scientific Corporation, San Diego, CA, USA), 9.5-µl water, 1-µl each of 10-µM forward and reverse primers, and 1 µl of DNA template according to the manufacturer’s protocol in a Veriti® thermocycler (Life Technologies, Carlsbad, CA, USA). The thermal cycler program for PCR was as follows: denaturation at 95 °C for 5 min, followed by 40 cycles of denaturation at 94 °C for 30 s, annealing at 55 °C for 45 s, and extension at 72 °C for 1 min. A final extension was performed at 72 °C for 10 min. PCR products were cleaned using ExoSap-IT (Affymetrix, Inc., Santa Clara, CA, USA) according to the manufacturer’s protocol. DNA sequencing was performed using PCR primers for direct sequencing by dideoxynucleotide chain termination using an ABI PRISM BigDye terminator cycle sequencing ready reaction kit (Life Technologies, Carlsbad, CA, USA) in an Applied Biosystems 3730 XL DNA Analyzer (Life Technologies) by the Genomic Sciences Laboratory (North Carolina State University, Raleigh, NC, USA). The molecular sequences were compared with other nematode species available at the GenBank sequence database using the BLASTn homology search program.

### Phylogenetic analyses

DNA sequences were edited with ChromasPro1.5 2003–2009 (Technelysium Pty Ltd, Helensvale, Australia) and were aligned by Mega7.0.14^64^ using default settings. The model of base substitution in the DNA sequence data was evaluated using MODELTEST version 3.06^65^. The Akaike-supported model^66^, the proportion of invariable sites, and the gamma distribution shape parameters and substitution rates were used in phylogenetic analyses using DNA sequence data. Bayesian analysis was performed to confirm the tree topology for each gene separately using MrBayes 3.1.0^67^, running the chain for 1,000,000 generations and setting the ‘burnin’ at 2,500. Markov Chain Monte Carlo (MCMC) methods were used within a Bayesian framework to estimate the posterior probabilities (pp) of the phylogenetic trees^68^ using the 50% majority-rule. The λ^2^ test for homogeneity of base frequencies and phylogenetic trees was performed using PAUP* version 4.0 (Sinauer Associates, Inc. Publishers, Sunderland, MA, USA).

### Species identification using PCR by species-specific primers

The species identification of *M. incognita* was confirmed using PCR by species-specific SCAR primers Inc-K14-F/Inc-K14-R which produce a 399-bp DNA fragment^69^. Another set of *M. incognita*-specific SCAR primers was a 1200-bp PCR fragment amplified by Finc/Rinc^21^. Fjav/Rjav^21^, Far/Rar^21^ and MH0F/MH1R^70^ were the other species-specific primers to *M. javanica*, *M. arenaria* and *M. hapla* which produced 670-bp, 420-bp and 960-bp DNA fragment respectively. The 25-µl PCR was performed using 12.5-µl 2X Apex *Taq* red master mix DNA polymerase, 7.5-µl water, 1-µl each of 10-µM forward and reverse primers, and 1-µl of DNA template. The PCR condition is the same as described above.
